# Exploring professional vision of vet students and tutors: noticing, evaluating and reasoning about practice

**DOI:** 10.1007/s12186-025-09375-4

**Published:** 2025-07-19

**Authors:** Sietse Brands, Maaike D. Endedijk, Bas Kollöffel, Elwin R. Savelsbergh

**Affiliations:** 1 Educational Science and Technology, University of Twente, De Zul 10, Enschede, NJ 7522 The Netherlands; 2 Research Group Curriculum Development in Primary and Secondary Education, University of Applied Sciences Utrecht, Utrecht, HU The Netherlands

**Keywords:** Workplace Learning, Workplace Guidance Vocational Education and Training (VET), Professional Vision, Knowledge-based Reasoning, Noticing

## Abstract

In dual vocational education and training (VET) programs, students learn across school and work contexts. Educators in both these contexts reside in different socio-cultural practices and can have different or even contradicting views on practice, influencing their guidance. In this study, we used the concept of professional vision to explore differing views on practice between participants with various educational roles, namely students, school teachers and workplace supervisors. In total, 11 students, 11 school teachers and seven workplace supervisors were asked to view the same set of five videos showing apprentices in the domain of underground infrastructure performing domain-specific tasks. Participants were asked to explain what they noticed in the videos and evaluate the apprentices’ performance. The data analysis consisted of a mixed-method approach. We quantitatively compared how educational roles viewed and evaluated task performance, followed by a qualitative ventriloquial analysis of their reasoning. Our findings highlight the diversity in professional vision among participants. Participants focused on different elements of practice, evaluated the same elements differently and showed variations in their reasoning. However, this could only be partly explained by their educational role. These findings suggest a need to prepare VET students for the different views of practice they will encounter during their careers, ultimately forming their well-founded professional vision.

## Introduction

Preparing for a profession is not only an individual process of developing knowledge and skills but also a social process (Illeris, 2003; Fuller, 2024). As learning to participate in multiple practices is crucial to becoming skilful professionals, students in dual vocational education and training (VET) programs alternate between school and workplace contexts. These contexts differ in terms of their culture, history, rules, and how they talk about practice (Akkerman & Bakker, 2011; Schaap et al., 2012; Tynjälä et al., 2021). Through interactions in the form of collaboration with experts, observations, explanations, discussions and feedback (Mikkonen et al., 2017), students integrate socio-culturally defined knowledge of the practice they are immersed in (Tynjälä et al., 2021). However, while traversing from one context to the other, students are confronted with different ways of working, norms, and values, sometimes complementary and sometimes conflicting (Akkerman & Bakker, 2011; Roth, 2014; Fuller, 2024). For example, different assessment methods and criteria are used (Sandal et al., 2014) as school teachers guide students towards passing central exams, abiding by formal protocols and assessment requirements, while efficiency and productivity are paramount at the workplace (Mikkonen et al., 2017). This may result in inconsistent feedback, confusion among students, feelings of discontinuity, interruption of their learning and feelings of wasting time (e.g., Roth, 2014; Fuller, 2024). At the same time, when learners are well-guided in making sense of these differences, these different perspectives also provide learning opportunities and help learners to develop an integrated knowledge base (Guile & Griffiths, 2001; Illeris, 2009; Tynjälä, 2008).

One of the major quests in vocational education and training is how to use the richness and diversity of the multiple contexts in dual forms of education to benefit instead of hampering the students’ learning processes. Scholars have suggested aligning and connecting school and workplace contexts in various ways to create a smoother contextual transition for students by improving collaboration between teachers and workplace supervisors (Hiim, 2023), creating boundary-crossing objects (Akkerman & Bakker, 2011), and hybrid integration of school and workplace through physical and digital collaborations resulting in new in-between practices (Bouw et al., 2019; Schwendimann et al., 2015). However, even the best-aligned VET practice requires students to deal with and integrate varying perspectives on practice. We propose that the concept of professional vision (Goodwin, 1994) is very well suited to understanding how students integrate these varying perspectives of the practices they are immersed in and subsequently form their perspective. However, most studies on professional vision have been limited to highly educated professionals (e.g., teachers and medical professionals) or strongly visually-oriented professions (Caruso et al., 2019) and often used a cognitive approach focusing on mental processes of noticing instead of also integrating a socio-cultural approach focusing on the situated nature of their professional vision (König et al., 2022).

To better understand how the concept of professional vision can help to make sense of differences in and between practices in dual VET programs, this study aims to get a better understanding of how different actors in dual VET programs, namely school teachers, workplace supervisors, and students, view and interpret workplace situations. We will systematically compare the professional vision of participants with different educational roles by showing them the same set of five videos in which apprentices perform domain-related tasks. We combine a socio-cultural (Goodwin, 1994) and cognitive approach (König et al., 2022) on professional vision to study *what* the participants *notice* in the videos and how they *interpret* what they notice. Second, we will use ventriloquial theory (Aveling et al., 2015; Nathues et al., 2021) to study the situated nature of their *reasoning*: what sources do participants with different socio-cultural backgrounds use to argue for their evaluations? The results of the study will contribute to our understanding of how socio-cultural differences and experiences contribute to professional vision development and provide pointers for the design of dual VET programs to ensure that the richness of the multiple practices benefits students’ learning.

### Professional vision: noticing, interpreting, and acting

Professional vision is a concept that sees ways of viewing and interpreting situations as socially organised; in other words, how people view and interpret a practice depends on their professional experiences and knowledge base (Goodwin, 1994). Professional vision is visible in the discursive practice of professionals and entails how they view and interpret concrete practice situations based on their social role, knowledge, experiences and culture (Goodwin, 1994). First, noticing revolves around the events one consciously or subconsciously chooses to pay attention to in a situation. Second, individuals make sense of things they notice using their prior experiences and knowledge base. Third, they decide how to act based on their interpretation (König, 2022). For example, when a teacher observes a student bowed over their workbook, a novice teacher might interpret this as hard-working, deciding to leave them alone. In contrast, expert teachers would use their experience to check if they bow to hide their gaze directed at their neighbour, revealing off-task behaviour that needs an intervention. Experts can pay attention to relevant information, while novices are unable to make this distinction, which appears to be a consistent finding across domains like tennis (Wood et al., 2023), navigating (Ooms et al., 2014), and analysing traffic situations (Anders et al., 2006). Studies on professional vision vary in their grounding and methods. While Goodwin’s initial professional vision theory is grounded in a socio-cultural approach, research gradually shifted towards a more cognitive approach, using quantitative methods focused on perceiving, reasoning and decision-making (König et al., 2022). Noticing has become a research theme on its own, in which researchers mainly aim to explore and train the noticing skills of novices (e.g. Rooney & Boud, 2019). Compared to the concept of noticing, which is more grounded in a cognitive-psychological perspective focusing on expertise as a primary factor explaining individual differences, the concept of professional vision strongly emphasises how they interpret situations in relation to their socio-cultural background (König et al., 2022). This makes professional vision an important indicator of the acquisition of integrated knowledge (Stürmer et al., 2015).

Studies that used the concept of professional vision have been conducted in various domains with various foci. Most studies on professional vision have been conducted in teacher education and from a cognitive approach. Novice-expert studies often concluded that novices greatly differ from experts in terms of viewing and interpreting classroom events due to a less developed knowledge base (Wolff et al., 2016, 2017). Beyond teacher education, the concept has been explored in fields like architecture, radiology, and healthcare. For instance, radiologists excel in detecting subtle anomalies in medical imaging, while architects use their expertise to interpret spatial layouts with both aesthetic and functional considerations. Recently, some research has been conducted in fields of lower VET levels. For example, Coppi et al. (2019, 2021) identified how professionals in diverse domains such as gardening, fashion design, and goldsmithing can be guided by visual tools to direct their observations and support their reasoning. Furthermore, Caruso et al. (2019) studied professional vision in the fashion domain and Gåfvels (2016) in floral design, both studies exploring domain-specific visual themes that individuals pay attention to.

Overall, scholars focused more on *what* is noticed and less on their *interpretation* of what they noticed, moving Goodwin’s original interpretation, including contextual, social, and cultural influences to the background. In this study, we adopt an integrated approach, as we are both interested in what people notice and in how they interpret it in relation to their expertise and their socio-cultural background. That means that we take a cognitive approach by focusing on what individuals notice in relation to their expertise (students versus teachers and supervisors) but also adopt the socio-cultural approach to understand how and why people interpret and reason about what they notice in relation to the practice they are grounded in (school versus workplace).

### Understanding socio-cultural differences in professional vision using multivoicedness

Following the integrative model of Tynjälä (2008), which emphasises the integration of theoretical knowledge, practical skills, and self-regulation to bridge the gap between school-based education and workplace learning, we postulate knowledge can originate from various sources, such as school teachers, colleagues from practice, or personal experiences. Although we know of the possible existence of this variety, a better understanding is needed of how people (both novices and experts) mobilise these sources when they notice and interpret what they see to explain differences in professional vision. To understand socio-cultural differences in professional vision, we use the concept of multivoicedness. Multivoicedness departs from the premise that the “Self” constitutes multiple dialogical voices: the voices of the self, the voices of others, and their interactions (Aveling et al., 2015). Individuals can thus speak from their preferences and experiences, but they can also voice other entities’ perspectives, opinions, rules, protocols, arguments, concerns, etcetera. For example, individuals could say: “I think this is very important”, or they could say “My colleagues highly value…” or “In line with this protocol, I…”. Unravelling how people give voice to other beings and make them speak - also called ventriloquism - originated in the field of organisational communication (Nathues et al., 2021), for example, to study various perspectives coming together during collaboration (e.g. Akkerman & Bakker, 2011; Nathues et al., 2023). However, it is also used to understand how apprentices build knowledge from both theoretical sources and experience (Renedo et al., 2018) and to study professional identity development, as these are also formed through negotiating multiple inner and other voices in relation to the work context (Arvaja, 2016).

As stated, students are prone to multiple perspectives and need to integrate them. When interpreting what they notice, they can voice multiple entities or perspectives with different weights (Aveling et al., 2015; Engstrand & Enberg, 2020). Therefore, part of dealing with different voices is concerned with an act of negotiating, a cognitive activity that occurs when individuals invoke contradicting voices (Nathues et al., 2021). For example, stating that two perspectives exist on a matter, but that in this specific case one is more applicable than the other. In their ethnographic study on learning workplace safety, Gherardi and Nicolini (2002) emphasised the importance of analysing language when understanding how novices become socialised in a new practice. They showed how operators use terms such as ‘you must’, ‘you cannot’ and ‘always’ when conveying tacit and explicit knowledge to novices. Additionally, operators used the voices of others (e.g., planning engineers or safety protocols) to invoke and negotiate the perspective of parties not actually part of the conversation. In conclusion, as different voices bring different perspectives to the world, we expect that the analysis of the voices participants invoke and, if and how they negotiate these voices to make sense of the practice situation they view, will help to understand socio-cultural differences in their professional vision.

### The current study

Differences in socio-cultural practice and experience greatly affect how individuals view and interpret practice situations. We expect the different socio-cultural practices of training schools and workplace contexts to affect how educators view and interpret the same practice. Also, students who participate as novices in both of these practices need to become acquainted with the different perspectives of their workplace and school teachers, make sense of these differences, and develop their professional vision. In this study, we explore differences in professional vision (what people notice and how they interpret this) between school teachers, workplace supervisors, and students. To do so, we adopt an approach in which teachers, supervisors, and students view and interpret a series of the same concrete instances of task performance. Our main research question is: “*How do school teachers*,* workplace supervisors*,* and students in dual VET programs in the domain of electrician education differ in what they notice and how they interpret domain-related task performance?*”

## Method

### Participants

This study was conducted in the context of Dutch dual electrician education at EQF levels 2 and 3. Students typically go to school one day and work as apprentices for the remainder of the week. Participants were recruited in three educational roles: Students, workplace supervisors, and school teachers. Students participating in the study were required to have at least three months of experience working in a simulation or authentic work environment. School teachers work at educational organisations and are charged with instructing procedures to students, often in a simulation environment. Workplace supervisors are electricians who supervise one or more apprentices daily at an authentic workplace. Participants were chartered using convenience sampling. Participants were informed about the research procedure and could either accept or decline to participate verbally. Our sample included eleven students, eleven school teachers and seven workplace supervisors. All participants were male, which is representative of this domain. The participants gave informed consent to participate in the study.

### Instrument and procedure

In one-on-one sessions with a participant and a researcher, each participant viewed the same five video recordings in the same order, each showing an apprentice’s performance of a domain-related task in an authentic or simulated environment. The five videos were not specifically selected for low- and high-quality performance. However, all were authentic videos capturing a part of a typical procedure in the domain (about a minute each). The first video started with a relatively simple task with only a few “mistakes” to give the participants an idea of what to expect. Four videos were recorded at the training school in a simulated environment, one at a workplace (Video 3). Videos were recorded using a head-mounted camera, providing a first-person view with audio of the practice situations (see Appendix A for pictures). In the videos, the performers orally explained what they were doing to the viewer. No other persons were involved in the video or audio. VET students recorded these videos as part of an experiment to work with a video-based (instead of written) portfolio. They consented to include the videos for this study’s purposes.

The first video showed a student performing a last-minute risk analysis before working indoors on a large consumer unit. In the video, the student’s preparation is visible; the tools are on the ground next to their working space, and a fire extinguisher is placed nearby. The student explains that a large cable is running on the ground that is not part of his work but that he cannot remove it before working. The second video showed a student working on a transformer. He creates an earth shield by twisting copper wires and then cutting them to attach them to the installation. The video showed the installation he is working on, and at the end of the video, the tool organisation becomes visible, showing many tools scattered on a plateau. The third video showed a student working in a pit outside. He is branching a cable under voltage to connect a house to the power grid. He is testing for voltage on the cable he has already worked on. He makes contact by jabbing their measurement instrument through the insulating layer of the cable. The fourth video showed a student working on a large indoor consumer unit. The student is in the process of attaching an earth shield and a large cable to the installation. The last video showed a student measuring voltage on an electricity meter that they will replace. He checks for voltage in various places in the installation and continuously checks whether their voltage meter works correctly.

During the data collection session, screen capture software recorded both the conversation and the video being viewed, allowing for analysis of what they were looking at and their interpretations simultaneously. Before viewing, participants were instructed to view the videos critically and to evaluate each performance, including their reasoning, as if they were to give feedback to a peer or student. They did not receive any support or rubrics to guide their attention. Participants were instructed to verbalise their thoughts and to think aloud about what they noticed. To gain more insight, the researcher asked questions to stimulate think-aloud or to understand their utterances better. Possible questions by the researcher were, for example, *“Where did you see that?”*,* “How do you know?”* or *“What do you think of this performance?”* After each video, the participants were asked to evaluate the performance and give arguments for their evaluation. The participants could review parts of the video recording as they wished.

### Data analysis

To analyse the participants’ professional vision, we focused on (1) what they noticed; (2) their evaluation of what they noticed; and (3) their reasoning behind it using a ventriloquial lens (Nathues et al., 2021). We compared the differences between the different educational roles in these three aspects.

In the first step, we identified what participants noticed and used these as the unit of analysis. Each element they mentioned was identified, and subsequent reasoning was included in these coding units. When a participant mentioned a topic multiple times, it was only coded once. Following Vásquez et al. (2018) we call the noticed topics ‘matters of concern’ (MoCs). An MoC could thus be something the performer did, like cutting a cable a certain way, or something visible in the environment or on the performer, like how the workplace was organised or the safety equipment the performer did or did not wear. An inductive analysis of all the mentioned topics resulted in a total of 56 unique MoCs over the five videos (see Appendix A for a distribution per video) that were in total mentioned 472 times, such as wearing fire or cut-resistant clothing, placing tools in certain spots, handling tools and materials, etcetera. The MoCs participants raised could be distinguished into three general themes. The three overarching themes we discerned were *personal protection equipmen*t, *organising and securing the workplace*, and *performing procedures* (see the Result section for detailed descriptions). In the second step, we analysed their interpretation of the MoCs. For each identified MoC in the first step, we aimed to identify whether the participant evaluated this as either positive, neutral or negative (see Appendix B for the codebook). For example, when an individual said, “he really needs to wear cut-resistant gloves here” or “This is clearly the wrong way of doing this”, these were coded as a negative evaluation. When a participant was in doubt and did not come to a conclusion, we coded the evaluation as neutral. An example is the following utterance: “I’m not sure if you need to wear insulating gloves or not. I usually wear cut-resistant gloves. But now I’m unsure about the regulations. I thought it wasn’t required.” Finally, positive evaluations were often straightforward, like “That’s how you’re supposed to do it. I think he’s doing well.”

In the third step, we studied individuals’ reasoning using ventriloquial analysis, distinguishing *personal* voices, *formal* voices, and *informal* voices (Aveling et al., 2015; Nathues et al., 2021) and an *unclear* category. For each identified MoC, we coded the voices used in their reasoning. We coded a personal voice when a participant speaks on their behalf, drawing from their own values, knowledge and experience. An example would be “I think this is wrong” or “This is nicely done”. These are accompanied by less directive verbs such as ‘*would*’. For example, ‘If I were him, I would do this or that’. Personal voices typically lack references to other perspectives or documents, e.g. peers’ opinions, examination requirements or protocols. When such references are present, the reasoning was coded as either ‘informal voice’ or ‘formal voice’. Voicing others typically entailed that the reasoning included a direct or indirect remark about a peer, tutor, or company. For example, a direct referral to another perspective would be ‘They often say you should do it like this’ or ‘My tutors told me to…’. The formal voice is similar in this regard, but instead of referring to others, it refers to more authoritative elements like examination requirements or safety protocols. Instances of the formal voice are ‘In the safety protocols, it is written to always do…’. Sometimes, references to these authoritative elements were more implicit, voicing that something is expected of the performer rather than something the individual participant would like to see. When voices are accompanied by directive verbs like ‘*must*’, ‘*should*’, and ‘*have to*’, it becomes apparent that this is the standard or a plain necessity. We coded this as implicit voicing of regulation, for example, ‘The fire-extinguisher must be located here’ or ‘The technician has to wear his insulating gloves during this procedure’.

Participants could invoke multiple voices on a MoC, but each type of voice was only coded once per MoC. Thus, for one MoC, the personal and informal voice could be invoked by a participant, but not two informal voices. If a participant mentioned two different voices, both were coded, and the most decisive voice was coded as the dominant voice. The dominant voice reflected the perspective that ultimately guided the participant’s evaluation regarding the MoC, regardless of whether it was introduced first or last in the discussion. This procedure was necessary to have the same unit of analysis (the MoC) for all analyses. The result section explicitly mentions when the dominant voice was used in an analysis. Additionally, when a participant mentioned different voices on a single MoC, this was coded as *negotiating*, indicating that the participant considered and weighed different perspectives on the MoC, before coming to a conclusion.

To check the quality of the codebook and coding procedure, all the data was coded independently by a second researcher. Interrater reliability was good, with a Cohen’s kappa of 0.82 for evaluations and 0.88 for voices. Any points of disagreement were discussed. As our sample sizes differed (11, 11, and 7), we used Kruskal-Wallis non-parametric tests to see whether there were significant between-group differences in the number of matters of concern the educational roles mentioned. To see how distributions differed between what is noticed, the evaluations, and invoked voices, we used chi-square tests with a Bonferroni correction.

## Results

In total, participants mentioned 472 MoCs (*M* = 16.28, *SD* = 5.61). On average, watching the videos and verbalising the MoCs took 18 min and 27 s (see Table 1). School teachers raised an average of 16.82 (*SD* = 5.96; duration 20:04 min) MoCs, students 12.91 (*SD* = 3.65; duration 15:09 min) and workplace supervisors 20.71 (*SD* = 4.57; duration 21:05 min) MoCs across the five videos. Kruskal-Wallis non-parametric tests indicate a significant difference in the average amount of MoCs between roles (H(2) = 8.702, *p* =.013). Follow-up pairwise comparisons using Dunn’s test showed a significant difference between students and workplace supervisors (*p* =.01) but not between other roles. This indicates that workplace supervisors raise on average more MoCs than students, but not more than school teachers. Nor do school teachers raise more MoCs than students.

**Table 1:** Distribution of matters of concerns and length of verbalisations across roles

			School teachers (*N* = 11)	Students (*N* = 11)	Workplace supervisors (*N* = 7)	Total
Theme	Protection	Count	28_a_ (15.1%)	39_b_ (27.5%)	39_b_ (26.9%)	106
		Expected Count	41.5	31.9	32.6	106.0
		Adjusted Residual	**−3.1**	1.7	1.5	
	Organisation	Count	52_a_ (28.1%)	37_a_ (26.1%)	42_a_ (29.0%)	131
		Expected Count	51.3	39.4	40.2	131.0
		Adjusted Residual	0.1	− 0.5	0.4	
	Procedure	Count	105_b_ (56.8%)	66_a_ (46.5%)	64_a_ (44.1%)	235
		Expected Count	92.1	70.7	72.2	235.0
		Adjusted Residual	**2.4**	− 0.9	−1.6	
Total		Count	185 (100%)	142 (100%)	145 (100%)	472 (100%)
		Expected Count	185.0	142.0	145.0	472.0
Length of Verbalisations		Minutes: Seconds (*SD*)	20:04 (*4:29*)	15:09 (*2:12*)	21:05 (*6:13*)	18:27

Each subscript letter denotes a subset of group roles categories whose column proportions do not differ significantly from each other at the 0.05 level

Adjusted residuals in **bold** deviate significantly from expected counts

### Noticing and raising matters of concern

In this paragraph, we first explain the three themes of MoCs and then zoom in the differences between participant groups in *what* they noticed.

#### Personal protective equipment

In total, 22% of the MoCs were related to personal protective equipment (*M* = 3.66, *SD* = 2.62). This concerns the use, misuse or lack of personal protection equipment. Personal protective equipment in electrician education is the equipment that practitioners need to wear to protect themselves from harm when working. The necessary equipment depends on the task at hand and can be distinguished in voltage protection, cut protection and fire protection. Voltage protection most saliently comes in the form of insulated gloves, but insulating boots and a rubber mat are also mentioned. When mentioning the need for cut protection when working with sharp objects, participants referred to wearing cut-resistant gloves and long sleeves. Protection against burns is ensured by wearing fire-retardant clothing and a face shield.

#### Organising and securing the workplace

The second MoC theme was how the workplace was organised and secured. This theme was identified in 28% of the cases (*M* = 3.38, *SD* = 2.00). Just like personal protective equipment, organising the workplace revolves around safety for a large part, but efficiency is also important. Participants mention creating a safe emergency route, thus not placing tools and materials directly in the path to safety. Related to this is creating a clean and clutter-free environment to minimise the risk of tripping or causing short circuits while working. Further, participants mentioned a more efficiency-based approach to organising the workplace, which is concerned with creating an efficient environment and storing tools and materials neatly. Creating an efficient environment is ambiguous and goes from placing tools on the side of the performer’s dominant hand to creating enough space to manoeuvre during the performance. A frequently mentioned efficiency aspect is how toolboxes and tools are organised and stored after use.

#### Performing procedures

About 50% of what was noticed concerned how procedures were performed (*M* = 8.10, *SD* = 2.89). Performing procedures in this domain was characterised predominantly by measuring the voltage on the installation that was going to be worked on. We could discern a preparation phase, where workers would expose live wires to measure. Then, the actual measurement itself, using the right tools correctly, measuring relevant elements in the correct order and finally, communicating the measurement results. Other instances of performing procedures were concerned with more pragmatic forms of safety. For example, using insulated tools when working on an installation under voltage or directly fixating loose cables after working on them to prevent forgetting to fixate them later. However, performing procedures could also relate to correct performance. Thus, in general, a large part of the MoCs were directed at working safely and maintaining correct performance. While it was not always possible to clearly distinguish between safety and efficiency, it appears that safety is the highest priority for most participants when viewing practice situations.

#### Differences in noticing and raising matters of concern

We found significant role differences in the number of specific themes mentioned (*χ*(4) = 10.554, *p* =.032). We see (see Table 1) that the distributions of what is noticed are quite similar for the students and workplace supervisors, but that the school teachers paid attention to different things. A closer look at the adjusted residuals (significant deviations in Bold) showed that school teachers raised significantly less protection and more procedure-related MoCs than students and workplace supervisors.

### Evaluations of matters of concern

Next, participants’ evaluations of MoCs were analysed. MoCs could be evaluated as positive, neutral, or negative. On average, participants rated most MoCs as negative (*M* = 8.55, *SD* = 3.61), followed by positive (*M* = 4.41, *SD* = 2.98) and neutral (*M* = 3.31, *SD* = 2.42) evaluations. The distribution of evaluations across educational roles is presented in Table 2, showing that this time, school teachers and students are quite similar to each other in their evaluations, while the workplace supervisor evaluated the MoCs differently (*χ*(4) = 17.229, *p* =.002). The adjusted residuals indicated that workplace supervisors less often evaluated an MoC as negative but more often scored a neutral or positive evaluation than students and school teachers. We wondered if this could also be explained by what was noticed, as school teachers more often focused on procedures, a theme that could be more prone to negative evaluations. We, therefore, conducted a layered Chi-square test to see if we would find similar differences when zooming in on their evaluations per theme. As Table 3 shows, the patterns are similar, with school teachers generally being more negative and workplace supervisors evaluating what they see as more neutral or positive. However, these differences were only significant for the evaluations within the organisational theme (*χ*(4) = 14.778, *p* =.005; See Table 3). We observed the same trends for the other two themes, but the results are not strong enough to be significant.

**Table 2:** Distribution of evaluations across educational roles

			School teachers (*N* = 11)	Students (*N* = 11)	Workplace supervisors (*N* = 7)	Total
Evaluation	Negative	Count	109_a_ (58.9%)	83_a_ (58.5%)	56_b_ (38.6%)	248
		Expected Count	97.2	74.6	76.2	248.0
		Adjusted Residual	**2.2**	1.7	**−4.0**	
	Neutral	Count	34_a, b_ (18.4%)	22_b_ (15.5%)	40_a_ (27.6%)	96
		Expected Count	37.6	28.9	29.5	96.0
		Adjusted Residual	− 0.8	−1.7	**2.6**	
	Positive	Count	42_a_ (22.6%)	37_a_ (26.1%)	49_a_ (33.8%)	128
		Expected Count	50.2	38.5	39.3	128.0
		Adjusted Residual	−1.7	− 0.3	**2.2**	
Total		Count	185 (100%)	142 (100%)	145 (100%)	472
		Expected Count	185.0	142.0	145.0	472.0

Each subscript letter denotes a subset of group roles categories whose column proportions do not differ significantly from each other at the 0.05 level

Adjusted residuals in **bold** deviate significantly from expected counts

**Table 3:** Evaluations of matters of concern across educational roles

			School teachers (*N* = 11)	Students (*N* = 11)	Workplace supervisors (*N* = 7)	Total
Protection	Negative	Count	13_a_	17_a_	9_a_	39
		Expected Count	10.3	14.3	14.3	39.0
		Adjusted Residual	1.2	1.1	**−2.2**	
	Neutral	Count	8_a_	6_a_	11_a_	25
		Expected Count	6.6	9.2	9.2	25.0
		Adjusted Residual	0.7	−1.5	0.9	
	Positive	Count	7_a_	16_a_	19_a_	42
		Expected Count	11.1	15.5	15.5	42.0
		Adjusted Residual	−1.8	0.2	1.5	
	Total	Count	28	39	39	106
Organisation	Negative	Count	32_a_	23_a_	13_b_	68
		Expected Count	27.0	19.2	21.8	68.0
		Adjusted Residual	1.8	1.5	**−3.3**	
	Neutral	Count	7_a_	4_a_	16_b_	27
		Expected Count	10.7	7.6	8.7	27.0
		Adjusted Residual	−1.6	−1.7	**3.4**	
	Positive	Count	13_a_	10_a_	13_a_	36
		Expected Count	14.3	10.2	11.5	36.0
		Adjusted Residual	− 0.5	− 0.1	0.6	
	Total	Count	52	37	42	131
Procedure	Negative	Count	64_a_	43_a_	34_a_	141
		Expected Count	63.0	39.6	38.4	141.0
		Adjusted Residual	0.3	1.0	−1.3	
	Neutral	Count	19_a_	12_a_	13_a_	44
		Expected Count	19.7	12.4	12.0	44.0
		Adjusted Residual	− 0.2	− 0.1	0.4	
	Positive	Count	22_a_	11_a_	17_a_	50
		Expected Count	22.3	14.0	13.6	50.0
		Adjusted Residual	− 0.1	−1.1	1.2	
	Total	Count	105	66	64	235
Total			185	142	145	472

Each subscript letter denotes a subset whose column proportions do not differ significantly from each other at the 0.05 level

We present some examples in the following excerpts to better understand these different perspectives. Excerpt A shows how a student and school teacher evaluated the same situation differently. The student reasons that he believes the workplace is not very well organised. However, he hurries to say that this may be their preference and that for others this may work. However, what is a not very well-organised workplace for the student was an organised setting for this school teacher, signalling how individuals’ reasoning about certain aspects of practice may greatly differ. In excerpt B, a similar situation unfolded where participants had differing views on whether gloves should be worn or not, and also with differing reasons. There is a student who is very decisive that gloves are a must in the situation, similar to some educators. But then there are educators who both think gloves are not required. A school teacher believes gloves are unnecessary when a certain distance is kept, while the workplace supervisor says the indicators on the instrument are decisive. Some MoCs invoked a wide variety of views and subsequent reasoning. We explore their reasoning further in the next section using a ventriloquial lens.

### Excerpt A. Two differing views on a MoC concerning a specific workplace organisation

**Table:** 

Student (male, second year): “Maybe he could have placed his tools a bit tidier. But that’s really a personal preference thing.” School teacher (male, senior): “It’s quite a clear layout. You can see his tools and it all looks pretty organised.”	

### Excerpt B. Five differing views on a protection-related MoC concerning wearing insulated gloves

**Table:** 

Student (male, second year): “He’s not wearing gloves, but he should. If he jabs into the cable he will short circuit it and receive a shock. Absolute knock-out.” School teacher (male, senior): “When to wear gloves and when not to… Normally, you just keep a distance of 5 centimeter from the voltage and you’re good.” School teacher (male, medior): “He could cause an electric arc if he misses the objects he want to measure. It’s very important for him to wear his gloves in this type of work.” Workplace supervisor (male, junior): “It’s not necessary to wear gloves here, because he stays in the safe zone behind the rings of his measurement instrument” Workplace supervisor (male, junior): “I would advise him to wear his insulated gloves in this particular situation.”	

### Reasoning about work practice using a ventriloquial lense

To understand why participant perspectives on practice do not always match, we explored further their reasoning about a situation. Our exploration found that participants raised different voices in their reasoning. As explained, the voice heard can be the individual’s own voice, showcasing the experience and values of this person. But individuals can also invoke the voice of others, vocalising values and ways of working of colleagues or of more authoritative entities, such as protocols and assessors. We describe the different voices that were found in participants’ reasoning. In our data, we found a clear difference between participants voicing their personal values or voices, that of peers and workplace culture, and finally, official documents and protocols. The personal (*M* = 7.55, *SD* = 3.87) and formal voice (*M* = 5.90, *SD* = 3.24) were, on average, used more often per person than the informal voice (*M* = 1.76, *SD* = 1.70). When the voice used was indistinct due to the reasoning lacking length or depth, we coded this as an unclear voice (*M* = 3.10, *SD* = 2.18).

#### The different voices raised

##### The personal voice

The own or personal voice was often heard when reasoning about practice. Typically, the own voice resonated a personal opinion, values or preferred way of working. In excerpt C, we present examples of participants speaking from their own voice. The way they talk shows that they speak from their own experience, for example, the pragmatic case that material in the trench may get dirty or that placing a toolbox next to the trench is more convenient.

### Excerpt C. Examples of own voices in pragmatic matters

**Table:** 

School teacher (male, medior): “It’s a narrow trench. The tools are placed in it as well. Put those on the side, you’ll have more room to work.” Workplace supervisor (male, medior): “Personally, I would not place the clamp in the trench. Basically if anyone passes by you end up with sand on your clamp.” School teacher (male, medior): “I would run this cable a bit longer if possible. It’s already pulling in the mantle, meaning there’s pressure there. I’m not sure if that was possible here, but it would run better.”	

#### The informal voice

When reasoning about practice, we found that participants in our sample often raised other voices. Thus, not only their own voice could be heard but also the voice of others. As mentioned, these voices do not necessarily directly belong to people but can also belong to things. In our sample, we identified several voices verbalised by the participants. The voices appeared to originate from their own and other socio-cultural practices, such as schools and workplaces.

Excerpt D shows an example of both a workplace and a school teacher raising the voice of socio-cultural practice common in their work environment. The school teacher describes how people are expected to work in practice, explaining that it is common only to receive one set of tools, referring with “they” to employers. The workplace supervisor states something similar, raising the voice of the organisation in which they were educated and the voice of common practice, or culture, in their region. Participants mentioning their mentors from practice were categorised as the informal voice instead of the formal voice, as from this excerpt, it becomes evident that the way of practice may significantly differ across organisations and individuals. In both these examples, it is clear that the participant negotiates the informal voice of others, weighing it against personal and socio-cultural values. We will explore the negotiation of voices in depth later.

### Excerpt D. Raising the voice of common socio-cultural practice

**Table:** 

School teacher (male, medior): “I really like to keep low-voltage tools exclusively for low-voltage work. For high-voltage work, you can use tools that aren’t insulated, as these activities are always voltage free. But that’s often not done in practice, and then they say: “Use your low voltage tools for everything”. I think that’s a pity because the insulation layer of tools is very frail and damages easily when you use these tools to quickly whack something in place.” Workplace supervisor (male, medior): “I can tell he doesn’t work in our region. It’s the way he twists the copper wires. I was taught at [organisation] that you have to braid the wires instead. It really shows. But it’s really where you were taught. Here they want it like this, and at another company they want it like that. But his method is not really wrong.”	

#### The formal voice

The third voice was categorised as the formal voice. The formal voice refers to standardised work protocols and regulations that electricians should abide by. The domain has strict regulations regarding safety, and manufacturers distribute specific instructions for installing all their materials. These protocols prescribe clear instructions on using personal protection equipment and the correct procedure for performing specific tasks. We found some instances in which individuals refer to these protocols directly and explicitly, as seen in excerpt E. It is clear these people refer to the protocols or exams. The mention of examinations is an important aspect of the formal voice, as the assessors of such exams are mainly required to follow strict assessment rubrics based on standardised protocols.

### Excerpt E. Referring to protocols

**Table:** 

Student (male, second year): “A rubber mat… He doesn’t use it. If you do that during an exam you will fail. And he is not wearing his gloves.” Workplace supervisor (male, medior): “I’m not sure if you need to wear insulating gloves or not. I usually wear cut-resistant gloves. But now I’m unsure about the regulations. I thought it wasn’t required.” School teacher (male, senior): “Yes, he was working according to the protocol. Testing his tool, switching off voltage […]. I have no remarks. I think it was right according to the regulations.”	

In most other instances where this voice was found, references to these protocols were more implicit. People often state something was done right or wrong, using directive and urgent verbs like “must” and “has to”. Also, using words like “actually” and “normally” indicates a standardised or normative way of working that should be applied. Examples are highlighted in excerpt F. Here, these school teachers do not explicitly mention regulations or protocols but have clearly defined norms for good practice. They talk with a lot of certainty and decisiveness. There appears to be a certain norm for these tasks that needs to be followed for an action to be considered correct. These voices differ from informal practices as the language is much more directive and assertive. Also, the word “we” is not present in such instances. Therefore, it appeared that not an organisation-specific perspective was voiced, but rather authoritative domain-broad regulation.

### Excerpt F. Implicitly voicing regulations, i.e. domain-specific standardised protocols or agreements

**Table:** 

School teacher (male, senior): “He checks his voltage meter. That is good. He must demonstrate the cable is voltage free. He checks his meter and then returns to the fuses and again. So actually that is too often.” School teacher (male, senior): “The same here. He tightens the cable lug correctly. He presses twice. Normally you have to do that three times.” School teacher (male, senior): “Yes, he checks his voltage meter way too often. But most importantly, he has to measure the rotary field. He didn’t. He wants to replace a meter and doesn’t measure a rotary field? Nonsense.”	

#### Comparing raised voices between roles

First, we used chi-square analysis to compare the participants’ dominant voices with the different educational roles raised. Results showed that there were indeed differences between the different types of participants (*χ*(6) = 13.608, *p* =.034). The adjusted residuals indicate that school teachers used more formal voices to reach their conclusions and less unclear voices than the other roles, as can be seen in Table 4.

**Table 4:** Distribution of dominant voices across roles

			School teachers (*N* = 11)	Students (*N* = 11)	Workplace supervisors (*N* = 7)	
Dominant Voice	Personal	Count	79_a_ (42.7%)	57_a_ (40.1)	62_a_ (42.7)	198
		Expected Count	77.6	59.6	60.8	198.0
		Adjusted Residual	0.3	− 0.5	0.2	
	Informal	Count	7_a_ (3.4%)	9_a_ (6.4)	10_a_ (6.9)	26
		Expected Count	10.2	7.8	8.0	26.0
		Adjusted Residual	−1.3	0.5	0.9	
	Formal	Count	76_a_ (41.5%)	42_a, b_ (29.6)	41_b_ (28.3)	159
		Expected Count	62.3	47.8	48.8	159.0
		Adjusted Residual	**2.7**	−1.2	−1.7	
	Unclear	Count	23_a_ (12.4%)	34_b_ (23.9)	32_a, b_ (22.1)	89
		Expected Count	34.9	26.8	27.3	89.0
		Adjusted Residual	**−2.9**	1.9	1.2	
Total		Count	185	142	145	472
		Expected Count	185.0	142.0	145.0	472.0

Each subscript letter denotes a subset of group roles categories whose column proportions do not differ significantly from each other at the 0.05 level

Adjusted residuals in **bold** deviate significantly from expected counts

#### Negotiating voices

As all participants must deal with different voices, we explored as a final step whether and how they negotiated these voices. This weighing, or rather negotiating, voices with different perspectives on practice allowed for a deeper insight into how individuals would evaluate practice. This negotiation of voices was relatively rare, only in 12.3% of the MoCs (*N* = 58). There were no significant differences between the roles (*χ*(2) = 5.167, *p* =.076) in how often students, teachers and workplace supervisors negotiated the different voices.

In excerpt G, two commonly negotiated MoCs are highlighted. Negotiation means weighing the authority and applicability of different voices or perspectives to the specific context. The first indicates the difference between protocols and a practical rule of thumb. The school teacher first mentions the issue and states that, in practice, it is deemed unnecessary to wear gloves in this situation. This is an example of the ‘informal voice’. However, they add the more formal voice of protocols, stating that from that view, it is highly advised and probably even required to wear gloves. By invoking both voices, they imply that they understand that this would be deemed safe in practice but that, for a formal assessment, this would not pass. The second utterance is a matter of braiding copper wires, which was mentioned and negotiated often. In this case, the school teacher acknowledges that different views on braiding co-exist but concludes that one is not necessarily better than the other. No formal voice was apparent in this case, which may be why this person did not come to one undisputed conclusion.

### Excerpt G. Explicitly voicing regulations, i.e. domain-specific standardised protocols or agreements

**Table:** 

School teacher (male, senior): You could wonder if he has to wear insulating gloves because he needs to keep a distance of five centimetres from voltage. He does. But in a trench like this… Most network operators and perhaps even safety regulations dictate that you need to wear gloves. School teacher (male, medior): He’s doing great. Regarding the earth shield, some people say you have to braid it. And then some people say to twist two wires around the earth shield. Braiding is difficult and not everyone can do it. Twisting neatly is also difficult and I think he does a good job. It’s whatever you want.	

## Discussion and conclusion

Students in dual vocational education and training (VET) programs shift between school and workplace contexts. In both contexts, different views on practice exist, and as a result, students are confronted with multiple and sometimes contradicting perspectives. In this paper, we used the concept of professional vision to identify and understand structural differences in how various actors from these programs view and interpret workplace situations. We aimed to answer the question: “*How do school teachers*,* workplace supervisors*,* and students in dual VET programs in the domain of electrician education differ in what they notice and how they interpret domain-related task performance?*” We operationalised professional vision as noticing and interpreting (evaluating and reasoning) professional task performance. We gathered data from school teachers, students and workplace supervisors who all observed the same five videotaped practices.

### Exploring the professional vision of practitioners

We found differences between roles in terms of what they noticed (matters of concern). Overall, students raised, on average, the fewest matters of concern (MoCs), followed by school teachers and workplace supervisors noticing the most. Students, as novices, have less experience in the field than their experienced educators and, therefore, may fail to notice some critical events in performance, a trend described in noticing (König et al., 2022; Rooney & Boud, 2019). Students especially raised organisation-related and procedural MoCs less often but did keep up with the workplace supervisors in terms of noticing personal protection-related concerns. This might indicate the multifaceted nature of professional vision (Caruso et al., 2019) resulting in a staged development. As in most programs, safety regulations are taught at the beginning of the program; this shows how students can become more proficient in noticing when their expertise in relation to the topic has matured.

Furthermore, although school teachers did not mention as many matters of concern as workplace supervisors, they tended to be more critical, particularly in their assessment of procedural matters. Conversely, workplace supervisors exhibited a more neutral or even positive stance towards certain aspects of performance, such as the organisation of workspaces and protective equipment. School teachers entrusted with imparting foundational knowledge and skills may prioritise adherence to standardised procedures and protocols. In contrast, workplace supervisors, immersed in practical tasks, may prioritise efficiency and productivity over strict adherence to formal regulations, and have a stronger focus on guidance than on summative assessment (De Vos et al., 2022). This outcome aligns with previous ethnographic studies (Gherardi & Nicolini, 2002; Roth, 2014), stating that people at work do not necessarily follow protocols to the letter but will look for ways to work more efficiently, neglecting safety protocols where they believe risks are small enough to justify a more efficient approach. As a result, workplace supervisors could be more understanding of the contexts presented in the video, allowing them to negotiate the necessity of wearing safety equipment and messy workplaces in specific situations.

Concluding, we see that the school teachers and workplace supervisors are most different from each other in *what* they see and *how they evaluate* this, with students being “in the middle”. Students are more similar to the workplace supervisor in *what* they notice, while they are very similar to the school teacher in evaluating what they see. This may reflect the varying perspectives and priorities of individuals occupying educational roles. School teachers, for instance, may be more attuned to procedural adherence and standardisation or improving student understanding, given their responsibility for imparting foundational knowledge and skills (Mikkonen et al., 2017). For similar reasons, students and workplace supervisors may emphasise safety and protective measures more, reflecting their immediate engagement with practical tasks and authentic scenarios. However, it is important to stress that *what* they notice and *how they evaluate* it are entangled. People embedded in different work cultures and with other educational roles naturally have different priorities and thus focus their attention on various elements of practice (Akkerman & Bakker, 2011). Therefore, school teachers who focused more on procedures also noticed the flaws in these procedures. At the same time, workplace supervisors, who were more focused on protection and organisation, saw that these specific themes were performed well. Following König et al. (2022), we therefore conclude that for understanding individual differences in professional vision, integrating the novice-expert dimension and the socio-cultural practices of the participants is paramount.

### Multivoicedness to study socio-cultural differences in professional vision

To further understand the origin of differences in what they noticed and how they evaluated this, we focused on their participants’ reasoning from the perspective of multivoicedness, using a ventriloquial lens (Aveling et al., 2015; Nathues et al., 2021). This way, we could discern the different sources people brought to the front for their reasoning. The personal voice was prevalent across all roles, reflecting individual opinions and preferences. The informal voice was used to describe instances where participants referred to others as sources, which was not often used. Finally, the formal voice, referring to official protocols and regulations, was significantly more pronounced among school teachers than in other roles. School teachers seemed closer to official regulations, theories and protocols than their counterparts at the workplace. Nevertheless, the workplace supervisors did not appear overly lenient in using an informal or personal voice either. Workplace supervisors, however, often presented reasoning that was not clearly vocalised from either their own or other voices, making it unclear what influenced their reasoning. A possible explanation is that workplace supervisors have internalised a particular way of working and developed a gut feeling about what good practice entails (Eraut, 2004). As guidance at the workplace is often indirect and insufficient resources are allocated (Mikkonen et al., 2017), this leads to limited time and pedagogical expertise. As a result, we could imagine that workplace supervision is focused less on explaining the reasoning behind such decisions than school teachers, who are more used to explaining theoretical and practical foundations underlying their views.

The ventriloquial analysis is an innovative way to capture the sources of participant reasoning brought to the front to back up their evaluation (Nathues et al., 2021; Vásquez et al., 2018). However, while this concept provides a good starting point for understanding differences in reasoning and the development of the professional vision, we postulate that it does not answer the question of the sources of participant knowledge. Theories of multivoicedness and ventriloquial analysis help to understand what is presented as part of the integrated self (personal voices) and when other voices are brought to the stage in reasoning (Aveling et al., 2015). Therefore, we did not explicitly ask people how they knew certain things and where these came from. In that regard, the negotiation of voices was an interesting indicator of how participants weighed their personal, formal and informal evaluation criteria. However, while interesting, we found only a limited presence of multiple voices. Therefore, future research could experiment with different elicitation techniques and longitudinal designs to see if explicit and implicit negotiation behaviour of voices as a foundation for professional vision development can be understood better.

### Supporting students’ development of professional vision amidst differing perspectives

In this study, not only differences between workplace supervisors and school teachers were studied. We also included students to understand how their professional vision might develop and differ from educators’ professional vision. Overall, it seems we captured a snapshot of student development towards full practitioners, as, in general, their professional vision hovers somewhere between the profile of school teachers and workplace supervisors. These findings correlate with Wolff et al. (2016, 2017) findings of how professional vision differs between experts and novices in teacher education, but that novice teachers are able to notice and provide reasoning for striking problematic events. Also, in our study, most students were often able to pinpoint major safety issues and explain why these were problematic. Contrarily, students were often unable to highlight more nuanced mistakes or performance deviations that supervisors did point out. Wolff et al. (2016) concluded that novices were more image-driven and experts were more knowledge-driven when viewing videos. We also observed that students often focused on the elements at the centre of the performance, where educators could also look for details in the background to identify more matters of concern. Moreover, our students’ reasoning appeared less grounded in formal voices than workplace and school teachers. This may indicate that students may have understood how to perform procedures but not yet understand why they should be performed specifically, highlighting that students’ critical thinking skills and knowledge base are less developed than their educators.

The present study raises important considerations for practice regarding the role of educational roles in shaping professional vision. The large variations between and within the groups of school teachers and workplace supervisors in evaluating performance stress how often students deal with differing perspectives. In addition, as workplace supervisors often did not clearly back up their reasoning, this could leave students in the dark about why specific procedures or safety measures need to be handled in a certain way. This underscores the need for dual VET programs to acknowledge and accommodate the diverse socio-cultural contexts in which individuals are and explicitly vocalise their reasoning. The concept of the multivoiced self is also used to study professional identity, providing insights into how people position themselves by negotiating their own (multiple) inner and other voices (Arvaja et al., 2016). This work also shows the reciprocity between one’s self and the work context and how misalignment can lead not only to disengagement but also to transformative development. Therefore, we suggest that educators recognise the learning opportunities and challenges that exposing students to various perspectives may bring. Through in-depth discussions and explicit negotiation of voices, students can be guided in the sense-making of various perspectives on practice. This enables students to develop their professional vision and a critical attitude towards the perspectives they may encounter in practice during their professional careers. The alignment of workplace and school practice is not just a matter of collaboration (Hiim, 2023; Nykänen et al., 2022); it is also a continuous discussion of ways of working and to explicate knowledge (Heusdens et al., 2016; Orozco et al., 2019) to help students make sense of varying perspectives (Jonasson, 2015).

### Study limitations

While our study offers valuable insights into professional vision within dual VET programs, several limitations may influence the interpretation and generalisability of our findings. Thus, they merit consideration in future research endeavours.

Firstly, our study’s experimental setting may have constrained our findings’ ecological validity. We aimed to standardise the stimuli across participants by utilising videos to depict workplace situations. However, this experimental setting may have elicited more focused attention and critical evaluation than would occur naturally in practice settings. Also, participants’ ability to scrutinise the videos through replaying, slowing, and pausing the footage may have inflated their level of scrutiny and criticality, potentially biasing their observations and evaluations. We did not analyse how the number of stops influenced their evaluations, but we recommend future studies to track this. At the same time, it was also a new and non-natural situation in which participants had to verbalise what they noticed in front of an unfamiliar researcher. In future studies, it would be interesting to elicit their thinking via peer conversations, creating - especially for the students - a safer environment in which to speak up.

Secondly, our study’s small sample size may limit the robustness and generalisability of our findings. Future studies with larger and more diverse samples are warranted to confirm and extend our findings. Also, a larger sample size would allow for a more in-depth analysis of the participants’ experience level and specific socio-cultural background.

Thirdly, we could only consider the matters of concern that the participants explicitly vocalised. For example, some participants might notice something that went well but not find it worth mentioning. We have experimented with eye-tracking to get more insight into what they noticed. However, as we used footage from a head-mounted camera, resulting in a moving target, eye-tracking data was impossible to analyse. For future studies, it would be interesting to repeat this study with a fixed camera position and include eye-tracking data to get more insights into what they notice in relation to their evaluations and reasoning.

Lastly, the explicit negotiation of voices from participants’ discourse may not fully capture the nuanced internal processes involved in reasoning and decision-making. Participants may have engaged in internal deliberations and negotiations before articulating their final evaluations and perspectives. Additionally, inexperienced participants may have mentioned some of the MoCs out of social desirability. Despite the researcher not being an expert in the field, it could be that novices made some educated guesses reflecting safety protocols. Even though their argumentation for their evaluations was asked, this could have influenced the results. Thus, more complex cognitive processes underlying professional vision formation might be involved that we have not all captured.

### Final conclusions

In conclusion, our study contributes to the ongoing discourse on the gap between theory and practice in dual VET by highlighting the nuanced differences in professional vision among individuals occupying different educational roles. By exploring the characteristics of professional vision in terms of noticing, evaluating and reasoning about performance, our findings provide valuable insights for the design of dual VET programs and interventions. We suggest that students’ professional vision development can be further enhanced by uncovering and discussing the concurrent differing views on practice in dual VET that students deal with daily. Critically analysing practice videos in classroom settings and discussing the ideas of good practice and discrepancies between professionals can be a good starting point for this. In sum, this study lays a foundation for future exploration into the complex dynamics of perspectives in dual VET, aiming to bridge the theory-practice gap and improve educational outcomes.

## Figures and Tables

**Table 5:**
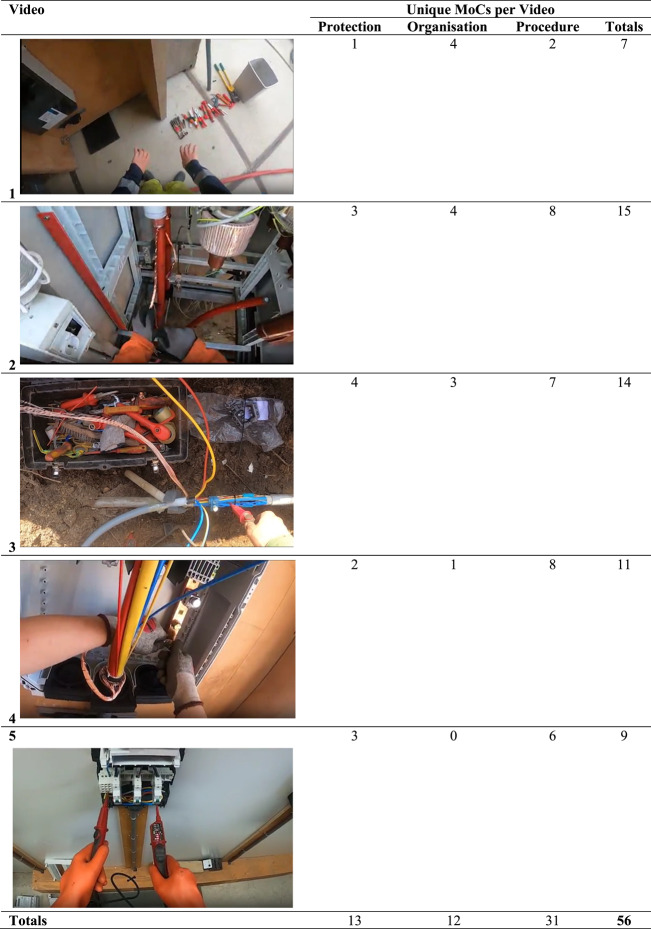
Unique Matter of Concerns (MoCs) per video, including a still from the video

## Data Availability

Data sharing is not applicable for this article due to the considerations for personal data and the participants’ declaration of consent.

## Appendix 2

**Table 6:** Coding scheme

**Theme**	**Code**	**Definition**	**Indicators**
Identifying Matters of Concern	MoC	Mentioned aspects of the recorded performance	Explicit mention of an aspect being noteworthy, problematic, or requiring attention.
Evaluations of Matters of Concern	Negative evaluation	A MoC is concluded to be incorrect.	Explicit rejection, phrases like"this is wrong."
	Neutral evaluation	No definitive conclusion, but curiosity or partial agreement.	Statements like"it’s not wrong, but…"or"I can’t see if he’s wearing his face shield, but let’s assume he is."
	Positive evaluation	A MoC is spoken about favorably.	Words like"neat"or"well done,"positive tone, absence of criticism.
Raising Voices	Personal voice	Reasoning based on own work style or perspective.	"I think,""I would,"references to personal preferences (e.g.,"easier,""faster").
	Other voice	References to others without explicit authority.	"They say,""in our region, we often do,""my supervisor says,"references to school teachings.
	Official voice	Explicit or implicit references to regulations, protocols, or authority.	"In principle,""must,"mentions of examination requirements or safety protocols (e.g., BEI guidelines).
	Unclear voice	Reasoning is too brief or ambiguous to determine perspective.	Short statements like"he is wearing gloves neatly,"lack of explanation for evaluation.
Negotiating Between Voices	Negotiating	Multiple voices are invoked and compared before forming a conclusion.	Weighing different perspectives before judgment, e.g.,"Ideally, grounding screens should be braided, but I also see other methods in practice."
